# Robust Real-Time DOA Estimation for Outdoor Vehicle Acoustic Sources Using Dynamic-Pruning GCC-PHAT and Adaptive Forgetting Factor OPAST-MUSIC

**DOI:** 10.3390/s26134281

**Published:** 2026-07-05

**Authors:** Xueheng Hu, Jianxin Zhang, Hong Ma, Jiaqing Shi, Yanyan Du

**Affiliations:** 1 School of Electronic Information and Communications, Huazhong University of Science and Technology, Wuhan 430074, China; d201780808@alumni.hust.edu.cn; 2 NORINCO Group Jiangshan Heavy Industry Research Institute Co., Ltd., Xiangyang 441057, China; xin5137@163.com (J.Z.); duyanyanzz@163.com (Y.D.)

**Keywords:** DOA estimation, DOA tracking, GCC-PHAT, OPAST-MUSIC, adaptive forgetting factor, dynamic pruning, microphone array, outdoor environments

## Abstract

In outdoor road environments, vehicle acoustic source direction-of-arrival (DOA) estimation is challenged by a low signal-to-noise ratio (SNR), dynamic-noise interference, and stringent real-time requirements. Under such conditions, conventional methods often struggle to achieve an effective balance among estimation accuracy, computational efficiency, and robustness against noise. To address this issue, this paper proposes a DOA estimation method that integrates a dynamic-pruning strategy with an adaptive subspace tracking mechanism. The proposed approach reduces computational complexity while enhancing algorithmic stability in complex and time-varying noise environments. Extensive experiments conducted on simulated data, the LOCATA dataset, and real-world outdoor road measurements demonstrate that the proposed method achieves comparable or superior DOA accuracy relative to conventional approaches, while significantly reducing computational cost. Furthermore, it exhibits stronger stability and robustness in real-world static and dynamic vehicle localization scenarios. Our method achieves a more favorable trade-off among multiple performance metrics. The results show that this method has good engineering application potential in complex outdoor environments, and can provide a practical solution for real-world vehicle monitoring.

## 1. Introduction

Among various sensing modalities, acoustic perception plays a crucial role. Acoustic feature monitoring possesses several inherent advantages, including passivity, concealment, and omnidirectional sensing capability. It is largely insensitive to terrain variations and visual occlusions and demonstrates strong adaptability to complex environments. Moreover, acoustic sensing is not affected by visibility conditions or electromagnetic interference, enabling reliable operation at night and in adverse weather conditions, such as fog and haze [1]. Consequently, microphone array-based acoustic sensing has become an important approach for outdoor environmental perception and monitoring.

In outdoor environmental monitoring, various time-sensitive and maneuvering ground targets generate characteristic acoustic signatures during motion, which can be captured and processed by acoustic sensors [2]. By employing acoustic sensing arrays, the time and frequency domain features of these signals can be analyzed. Combined with collaborative detection and recognition algorithms that incorporate target classification, localization, and trajectory estimation, it is possible to achieve comprehensive situational awareness, including target perception, identification, DOA estimation, and localization.

Truck acoustic sources are characterized by low fundamental frequencies and wide bandwidths, with dominant spectral components even below 100 Hz. However, the aperture and the number of microphones in a practical array are inherently limited, restricting the achievable array diameter. Although low-frequency acoustic signals exhibit relatively stable source characteristics, their long wavelengths make precise DOA estimation challenging when using compact microphone arrays. In contrast, the energy of higher-order harmonics decreases as the harmonic order increases, accompanied by reduced stability. In outdoor environments, these high-frequency components are more susceptible to masking by background noise, which significantly limits their effectiveness for reliable acoustic localization.

When trucks operate in outdoor environments, their acoustic emissions are inevitably mixed with ambient noise, resulting in a harsh operating condition for DOA estimation algorithms characterized by low SNR and strong interference. Moreover, the motion trajectory and velocity of the target are generally unpredictable, leading to a nonuniform rate of change in the relative bearing angle with respect to the microphone array [3]. Under such conditions, conventional recursion algorithms with fixed step sizes are often inadequate to accurately track the time-varying DOA, limiting their practical effectiveness in dynamic scenarios [4].

In summary, this paper proposes a recursion and pruning-based microphone array localization method for outdoor dynamic acoustic sources. The main contributions can be summarized as follows:

(1) A recursion and pruning-based far-field sound source DOA estimation framework is developed according to the characteristics of the target application scenario. The proposed method enables low-frequency and dynamically varying sound source localization using a compact microphone array.
(2) Within the generalized cross-correlation (GCC) framework for microphone arrays, a weighting matrix constructed based on inter-microphone pair distance and incident angle is introduced to perform dynamic pruning and recursion updating. This design reduces computational complexity while mitigating error-induced perturbations.
(3) For the multiple signal classification (MUSIC) algorithm, an adaptive forgetting factor-based recursion scheme is incorporated to balance the influence of varying angular velocity dynamics, thereby improving tracking stability under nonuniform target motion.

## 2. Related Work

Sound source localization based on time delay estimation (TDE) represents a classical methodological framework for acoustic localization utilizing microphone arrays. Among the various approaches within this framework, the GCC method, introduced by Knapp and Carter, enhances the robustness of time delay estimation in noisy environments through the incorporation of frequency domain weighting functions [5]. Subsequently, Benesty et al. improved the temporal resolution of time delay estimation by employing linear interpolation on the cross-correlation function [6].

Building on these advancements, researchers have further combined GCC with beamforming techniques, leading to the development of the steered response power (SRP) framework for sound source localization. Cobos et al. introduced an enhanced SRP-PHAT formulation to improve scalability for large-scale microphone arrays [7], whereas Do et al. presented a stochastic region contraction (SRC) strategy aimed at reducing the computational complexity of SRP-PHAT in large array search spaces [8].

Additionally, Salvati et al. enhanced the robustness of SRP in broadband noisy settings by employing frequency fusion techniques [2] and further introduced optimized spatial sampling grids to improve localization efficiency [9]. In multi-source scenarios, Blandin et al. utilized angular spectrum analysis in conjunction with clustering algorithms to achieve multi-source time difference of arrival (TDOA) estimation, thereby enhancing localization performance in reverberant environments [10].

High-resolution DOA estimation constitutes a pivotal research area within the realm of array signal processing. The MUSIC algorithm, introduced by Schmidt, leverages the orthogonality between the signal subspace and the noise subspace to attain high-resolution DOA estimation [11]. To tackle scenarios involving broadband signals, Yoon et al. proposed the TOPS algorithm, which accomplishes broadband DOA estimation through subspace projection [12], whereas Hung and Kaveh advocated the utilization of focusing matrices to harmonize subspaces across diverse frequency bins [13]. In intricate noise environments, the ROC-MUSIC algorithm, put forth by Tsakalides and Nikias, augments robustness against impulsive noise [14]. Moreover, Visuri et al. enhanced the performance of subspace-based DOA estimation by integrating nonparametric statistical methods [15].

In addition, array structure optimization has been extensively employed to improve DOA estimation performance. For example, Mathews and Zoltowski explored the problem of two-dimensional angle estimation within the context of a uniform circular array [16], whereas Liao and Chan presented a direction estimation algorithm tailored for partially calibrated arrays [17]. Pal and Vaidyanathan introduced a nested array configuration [18], Qin et al. proposed a generalized coprime array structure [19], and Liu and Vaidyanathan introduced a super-nested array [20]. Additionally, Wang and Nehorai conducted a theoretical analysis of the performance of the conventional array MUSIC method along with its corresponding Cramér–Rao lower bound [21].

To address the high computational complexity of subspace algorithms, Yang proposed the projection approximation subspace tracking (PAST) algorithm, which can recursively update the signal subspace, thereby avoiding repeated eigenvalue decomposition [4], and further conducted a theoretical analysis of its asymptotic convergence [22]. Based on this, Abed-Meraim et al. proposed the orthogonalized OPAST algorithm, which improves algorithm stability by maintaining the orthogonality of the subspace basis vectors [23]. In addition, Miao and Hua proposed a fast subspace tracking method based on information criteria [24], as well as the FAPI algorithm [25], YAST algorithm [26], and other subspace methods [3,27]. These methods significantly reduce the complexity of subspace updating, enabling subspace-based DOA estimation algorithms to be applied in real-time systems.

In practical scenarios, Valin et al. presented a multi-sound source tracking approach that integrates beamforming with particle filtering [28], whereas Huang et al. introduced a passive sound source localization technique tailored for video systems [29]. In recent years, several studies have implemented sound source localization systems on unmanned aerial vehicle (UAV) platforms, exemplified by the UAV acoustic perception system proposed by Salvati and Daniele [30] and the UAV-embedded microphone array developed by Hoshiba et al. [31]. Additionally, within wireless sensor network environments, Song et al. proposed a sound source localization system utilizing a distributed microphone array [32].

To address the challenge of vehicle sound source localization in traffic scenarios, Zhang et al. constructed a vehicle whistle database and investigated outdoor positioning performance [33]. Marques et al. explored acoustic detection and positioning systems within autonomous vehicles [34], while Marciniuk et al. employed machine learning techniques for traffic noise monitoring and localization [1].

In recent years, deep learning and sparse representation methods have also been applied to DOA estimation. Chakrabarty et al. employed convolutional neural networks (CNNs) for broadband DOA estimation, thereby improving robustness in complex noisy environments [35]. He et al. used deep neural networks to achieve multi-source detection and localization [36]. The SPICE and LIKES methods were developed by Stoica and Babu [37], and the sparse Bayesian DOA estimation algorithm was presented by Yang et al. [38].

In addition, deep learning-based approaches have been successfully applied to scenarios such as unmanned aerial vehicle acoustic source localization and cross-environment DOA estimation [39,40], demonstrating favorable robustness and generalization capability. Zhang et al. proposed a self-supervised cascaded deep neural network DOA estimation method, which effectively improved the localization performance under array error conditions [41].

In summary, existing studies have made substantial progress in array-based sound source localization algorithms and application systems. However, for outdoor far-field vehicle DOA estimation, two key challenges remain. First, under multi-microphone array configurations, GCC- or SRP-based methods require the computation of a large number of inter-microphone cross-correlation functions, leading to high computational complexity. Second, conventional MUSIC algorithms rely on eigenvalue decomposition of the covariance matrix, making it difficult to achieve efficient real-time processing in dynamic scenarios. Therefore, it is necessary to investigate the integration of low-complexity cross-correlation computation methods with real-time subspace tracking algorithms in order to improve both the computational efficiency and robustness of outdoor vehicle sound source localization systems.

## 3. Methods

### 3.1. Problem Definition

For microphone array-based sound source localization of outdoor heavy vehicles, the main challenges can be summarized as follows:

(1) The target acoustic source is characterized by a wide frequency band with multiple dominant spectral components. However, the microphone array aperture is relatively small, leading to a significant mismatch between the wavelength of the dominant low-frequency components and the array size. As a result, accurate localization based on low-frequency components is difficult, while direction estimation using high-frequency harmonics tends to be unstable due to reduced signal energy and increased susceptibility to noise.
(2) Due to constraints in mission duration, power consumption, and system cost, the onboard computational resources of the localization device are limited. Meanwhile, the amount of signal data increases with the number of microphones, making it necessary to balance algorithmic complexity and real-time performance. Otherwise, real-time tracking of dynamic targets cannot be achieved in practical scenarios.
(3) The target is a moving acoustic source with continuously varying positions and operating in complex outdoor noise environments. High-accuracy prediction is required for localization tasks; however, in such conditions, there exists an inherent trade-off between real-time performance, stability, and estimation accuracy, which poses significant challenges for algorithm design.

### 3.2. Overall Structure of Algorithm

Based on the aforementioned challenges, this study proposes the following solution framework for heavy vehicle sound source DOA estimation in outdoor scenarios:

(1) The acoustic characteristics of the target source are first analyzed, and both the dominant fundamental frequency and higher-order harmonic components are identified. Different processing strategies are then applied to these components accordingly.
(2) The GCC-PHAT method is employed to process the low-frequency dominant signal components, while the MUSIC algorithm is applied to the higher-order harmonic components for DOA estimation.
(3) Both algorithms are further optimized. A pruning-based GCC-PHAT strategy is adopted to ensure that the computational complexity does not increase with the number of microphones, thereby maintaining real-time performance and improving stability. Meanwhile, an adaptive recursive MUSIC algorithm is introduced to reduce computational cost while preserving high estimation accuracy.
(4) Finally, the two methods are integrated in a cascaded framework. The recursive pruning-based GCC-PHAT method provides a coarse initial DOA estimate and constrains the search space of the adaptive recursive MUSIC algorithm. Subsequently, the MUSIC-based method performs fine-grained DOA estimation within the reduced search region, achieving both efficiency and high precision.

**Figure 1:**
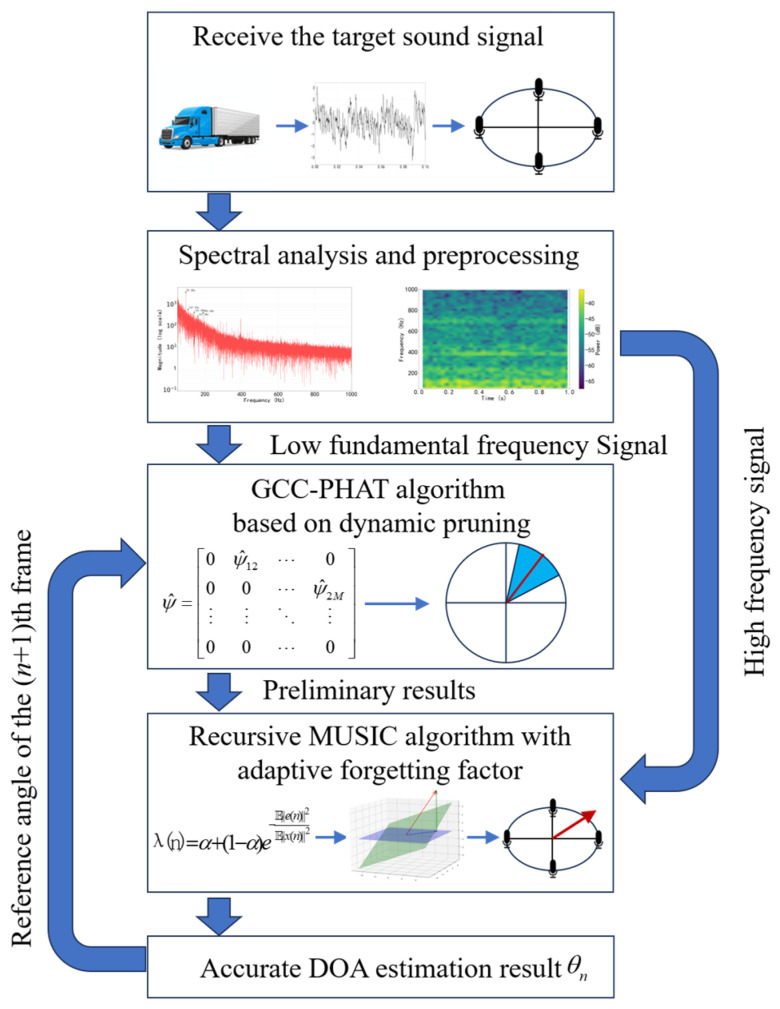
Overall structure diagram of algorithm.

As illustrated in Figure 1, the procedure of the proposed algorithm can be summarized as Algorithm 1:

**Algorithm** **1**: DOA Estimation based on Dynamic Pruning GCC-PHAT and Adaptive Forgetting Factor OPAST-MUSIC**Input**: DOA estimation $\theta_{n-1}^{music}$ at (*n* − 1)-th frame, pruning retention number *P*.1: **While** the microphone array receives the *n*-th frame signal:2:    The *n*-th frame signal is analyzed and preprocessed to extract its spectral characteristics.   The fundamental frequency and higher order harmonics are identified, and band pass    filtering is applied to isolate the corresponding frequency bands.3:    **If** *n* ≠ 1:      The DOA estimate obtained from the (*n* − 1)-th frame, denoted as $\theta_{n-1}^{music}$, is used as      the predicted incident angle of the target source. Based on this prediction, a      dynamic masking weight matrix is constructed to prune the cross-correlation matrix.      Then, using the pruned *P* pairs, a rough DOA estimation is performed on the      cross-correlation matrix composed of microphones to obtain the initial estimate $\theta_{n}^{gcc}$.4:    **Else**:      In the initial DOA prediction, *P* pairs of microphones are randomly selected to      perform rough DOA estimation on the sound source, resulting in $\theta_{1}^{gcc}$.5:    The value $\left[\theta_{n}^{gcc}-\zeta ,\theta_{n}^{gcc}+\zeta \right]$ is used to constrain the search range of the adaptive forgetting   factor–based recursive MUSIC algorithm for the *n*-th frame. The high frequency   harmonic components obtained in step (2) are then processed using adaptive subspace   tracking with a time-varying forgetting factor, resulting in a refined DOA estimate $\theta_{n}^{music}$.   This refined estimate is taken as the final DOA result for the current frame and is   subsequently used as the predicted angle for pruning in the (*n* + 1)-th frame.**Output**: DOA estimation $\theta_{n}^{music}$ for the *n*-th frame.

### 3.3. Dynamic Pruning-Based GCC-PHAT Algorithm

In the GCC algorithm, global spatial spectrum fusion is typically performed using a homogeneous equal-weight summation strategy. This approach largely ignores the nonlinear constraints imposed by the physical geometry of the microphone array on DOA estimation accuracy. Moreover, in multi-microphone array configurations, GCC-based localization does not necessarily require the full set of microphone pairs, as reliable direction estimation can often be achieved using only a subset of them.

Specifically, the localization error associated with individual microphone pairs is not uniformly distributed in space. When the incident acoustic wave arrives from the end-fire direction of a microphone pair (i.e., near-parallel propagation relative to the baseline), small perturbations in time delay estimation are nonlinearly amplified by the inverse cosine mapping, leading to severe error magnification. In addition, microphone pairs with excessively short baselines provide insufficient effective spatial sampling, resulting in increased estimation uncertainty. If such geometrically unfavorable and noise-sensitive cross-correlation measurements are indiscriminately incorporated into the global cost function, they inevitably lead to spatial spectrum broadening, the emergence of spurious peaks, and a significant degradation in global localization variance.

To address this fundamental limitation, a dynamic weight-pruning strategy based on baseline length and estimated local incidence angle is introduced to enhance the GCC-PHAT framework. This mechanism constructs a spatially aware dynamic masking matrix to quantify the geometric reliability of each microphone pair in real time. Only the highest confidence pairs are selected for DOA estimation, while the remaining pairs are explicitly discarded via hard pruning (i.e., their weights are set to zero). In this way, the propagation of geometric errors into the global estimation process is effectively suppressed at both the physical and mathematical levels.

This improvement not only preserves the inherent robustness of GCC-PHAT against reverberation, but also fundamentally eliminates spatial artifacts and nonlinear variance effects, leading to a significantly sharper spatial spectrum. As a result, both angular resolution and absolute localization accuracy are improved in complex and nonideal acoustic environments. Furthermore, since only a subset of microphone pairs is activated for computation, the overall computational complexity is substantially reduced and becomes independent of the total number of microphones.

#### 3.3.1. DOA Estimation Error Analysis for a Dual-Microphone Array

Consider a pair of microphones separated by a distance *d*. The line connecting the two microphones is defined as the 0° reference direction, while its normal direction is defined as 90°. A far-field acoustic source is assumed to impinge on the array with an incident angle *θ*. Let the TDOA be denoted by *τ*, and the speed of sound by *c*. Under these assumptions, the TDOA can be expressed as follows:

(1) $$\tau (\theta )=\frac{d\cos(\theta )}{c},\;\theta =\arccos(\frac{c\tau }{d})$$

The GCC-PHAT algorithm estimates the DOA of the sound source by first estimating the time delay. In this process, estimation errors are inevitably introduced, which can be expressed as follows:

(2) $$\hat{\tau }=\tau +\Delta \tau$$

where $\hat{\tau }$ denotes the estimated TDOA, τ represents the ideal time delay, and Δτ corresponds to the estimation error. In practice, a physical localization system cannot access the ideal time delay and can only obtain a noisy measurement, denoted as $\hat{\tau }$, which inherently contains estimation errors.

Taking the derivative of Equation (2), we obtain

(3) $$\frac{\partial \hat{\tau }}{\partial \theta }=-\frac{d\sin(\theta )}{c}$$

Taking the absolute value of Equation (3), the angular error amplification factor can be expressed as

(4) $$\Delta \theta \approx \left|\frac{\partial \theta }{\partial \hat{\tau }}\right|\cdot \Delta \tau =\left|\frac{c}{d\sin(\theta )}\right|\cdot \Delta \tau$$

where Δθ denotes the angular estimation error, c/d represents the array scaling factor, and |sin(θ)| corresponds to the angular-dependent factor. It can be observed that, under otherwise identical conditions, the primary factors influencing the estimation error of the GCC-PHAT algorithm are the incident angle of the sound source relative to the microphone pair and the inter-microphone spacing.

**Figure 2:**
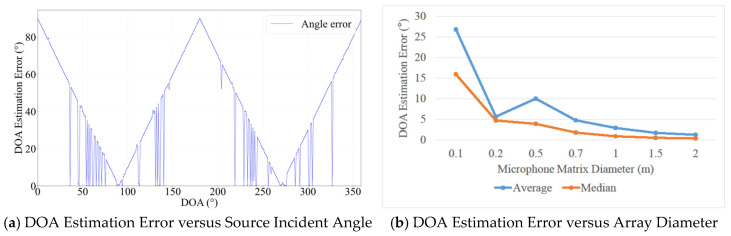
Error Analysis of Dual-Microphone Array DOA Estimation.

In Figure 2a, the horizontal axis represents the true DOA of the sound source, and the vertical axis represents the error of DOA estimation. It can be observed that, apart from a few outliers caused by noise, the DOA estimation error generally increases as the incident angle of the sound source approaches the array baseline. Specifically, for a sound source with a fixed frequency, when the diameter of the microphone array exceeds its theoretical upper bound, we have

(5) $$d_{\max}=\frac{\lambda_{\min}}{2}=\frac{c}{2f_{\max}}$$

When the inter-microphone spacing reaches *d* > *d*_max_, spatial aliasing occurs, leading to multiple spurious peaks with similar amplitudes in the generalized cross-correlation function, making it difficult for the algorithm to identify the true time delay. When the microphone spacing is below this upper bound, increasing the inter-microphone distance results in a larger time delay under a fixed sampling rate, thereby increasing the number of effective delay samples and improving estimation accuracy. In addition, as *d* increases, c/d decreases; consequently, the system time delay error Δτ also decreases.

As shown in Figure 2b, under otherwise identical conditions, when the diameter of the microphone array remains within the allowable upper bound, the DOA estimation error of the GCC-PHAT algorithm decreases as the array aperture increases.

Regarding the source incident angle, |sin(θ)| in Equation (4) indicates that when the incident angle θ approaches 90°, the value of 1/|sin(θ)| reaches its minimum, resulting in the smallest estimation error of the algorithm. Conversely, when the incident angle approaches 0° or 180°, 1/|sin(θ)| tends toward infinity, leading to significant error amplification. Therefore, it can be concluded that the DOA estimation error is minimized when the source arrives along the direction normal to the microphone axis, whereas it becomes significantly larger when the source direction is aligned with the array baseline.

Therefore, it is necessary to perform dynamic pruning of the generalized cross-correlation matrix according to the geometry of the microphone array and the estimated incident angle of the current target. Only the most informative and geometrically reliable microphone pairs are selected to participate in the DOA estimation at the current time step.

#### 3.3.2. Dynamic Pruning-Based GCC-PHAT DOA Estimation Algorithm

For multi-microphone arrays, the DOA estimation errors vary across different microphone pairs, and only a subset of these pairs is typically sufficient to achieve reliable localization. For compact arrays, microphone pairs with longer baselines tend to provide more accurate time delay estimates when the source incident angle is approximately perpendicular to the line connecting the pair. In such cases, the GCC-PHAT method yields lower TDOA estimation errors, thereby contributing positively to the overall array localization performance. Conversely, when the incident angle deviates significantly from this configuration, the corresponding microphone pair exhibits larger time delay estimation errors, which may introduce additional uncertainty into the overall DOA estimation results of the array.

The GCC-PHAT algorithm for a multi-microphone array can be formulated as follows:

(6) G(w)=W(w)Φ(w)

where G(w) denotes the generalized cross power spectral density matrix, W(w) represents the weighting function matrix, and Φ(w) corresponds to the signal power spectral density matrix. All three matrices have dimensions (M,M). Specifically,

(7) $$G_{ij}(w)=W_{ij}(w)\Phi_{ij}(w)$$

where $G_{ij}(w)$ denotes the generalized cross power spectral density (CPSD) between microphone i and microphone j. If the distance between microphones i and j is $d_{ij}$, then the corresponding array length-based weighting term is given by $D_{ij}$.

(8) $$D_{ij}=d_{ij}/Max(d_{mn})$$

where $d_{ij},d_{mn}\in d$, *d* is the set of distances between all microphone pairs, and $Max\left(d_{mn}\right)$ represents selecting the largest element in *d*, $D_{ij}\in \left[0,1\right]$. Then, the distance weighting matrix *D* of the entire array can be expressed as

(9) $$D=\left[\begin{array}{cccc} D_{11} & D_{12} & \cdots & D_{1M}\\ D_{21} & D_{21} & \cdots & D_{2M}\\ \vdots & \vdots & \ddots & \vdots \\ D_{M1} & D_{M2} & \cdots & D_{MM} \end{array}\right]$$

For the incident angle weighting matrix, the source incident angle for each microphone pair can be derived based on the estimated DOA of the sound source. In far-field source localization, regardless of whether the source is static or moving, the incident angle of a given source does not change abruptly; that is, the DOA varies smoothly between consecutive frames. Therefore, in continuous localization, the DOA estimate from the $\left(n-1\right)$-th frame can be used as prior information to guide dynamic pruning in the n-th frame.

For a dual-microphone array, if the source incident angle at the $\left(n-1\right)$-th frame lies within a low-error angular region, it is highly likely that the incident angle at frame n will remain within the same low-error region. Based on this observation, appropriate microphone pairs can be selected accordingly for DOA estimation.

At time n, consider a dual-microphone array located in a two-dimensional Cartesian coordinate system. The global incident angle of the sound source estimated at frame n−1 is denoted as θ(n−1) and θ(n−1)∈[−π,π]. The unit direction vector of the sound source is given by ${\left[\cos(\theta (n-1)),\sin(\theta (n-1))\right]}^{H}$. The coordinates of microphones i and j are denoted as ${\left[x_{i},y_{i}\right]}^{T}$ and ${\left[x_{j},y_{j}\right]}^{T}$, respectively. The baseline connecting the two microphones is, therefore, represented as ${\left[x_{j}-x_{i},y_{j}-y_{i}\right]}^{T}$, and the global orientation angle of this baseline can be calculated as

(10) $$\alpha_{ij}=\mathrm{atan}2(y_{j}-y_{i},x_{j}-x_{i})$$

where $\alpha_{ij}\in [-\pi ,\pi ]$. From a geometric perspective, the difference between the global incident angle θ(n−1) and the baseline orientation angle $\alpha_{ij}$ corresponds to the theoretical local incident angle “perceived” by the microphone pair, denoted as $\phi_{ij}^{raw}(n)$:

(11) $$\phi_{ij}^{raw}(n)=\theta (n-1)-\alpha_{ij}$$

Since both θ(n−1) and $\alpha_{ij}$ lie within the range $\left(-\pi ,\pi \right]$, their difference $\phi_{ij}^{raw}\left(n\right)$ may fall outside the interval $\left(-2\pi ,2\pi \right]$. Therefore, angle normalization is required.

(12) $$\phi_{ij}^{raw}(n)=atan2(sin(\theta (n-1)-\alpha_{ij}),cos(\theta (n-1)-\alpha_{ij}))$$

where $\phi_{ij}^{raw}(n)\in [-\pi ,\pi ]$.

(13) ϕij(n)=1−||ϕijraw(n)×180π|−90|90

From Equation (13), it can be observed that the closer the absolute value of $\phi_{ij}^{raw}(n)$ is to 90°, the larger the value of $\phi_{ij}(n)$; conversely, as it moves farther away from 90°, the value of $\phi_{ij}(n)$ decreases. For the microphone array, the angular weighting matrix can be expressed as

(14) $$\phi (n)=\left[\begin{array}{cccc} \phi_{11}(n) & \phi_{12}(n) & \cdots & \phi_{1M}(n)\\ \phi_{21}(n) & \phi_{21}(n) & \cdots & \phi_{2M}(n)\\ \vdots & \vdots & \ddots & \vdots \\ \phi_{M1}(n) & \phi_{M2}(n) & \cdots & \phi_{MM}(n) \end{array}\right]$$

The fusion of the distance matrix and the angular weighting matrix can be expressed as

(15) ψ(n)=D+ϕ(n)

where ψ(n) denotes the pruning weight matrix and each element represents the scoring weight of a microphone pair within the array. $\psi_{ij}(n)=D_{ij}+\phi_{ij}(n)$, the matrix ψ(n), has dimensions (M,M). Considering the symmetry of microphone pairs ($\psi_{ij}(n)=\psi_{ji}(n)$), only the upper triangular part of the matrix ψ(n) is retained, selecting elements $\phi_{ij}(n)$ that satisfy i,j∈[1,2…M] and i<j. The resulting formulation can be expanded as follows:

(16) $$\hat{\psi }(n)=[{\hat{\psi }}_{12}(n),{\hat{\psi }}_{13}(n),\ldots {\hat{\psi }}_{\left[M-1,M\right]}(n)]$$

where $\hat{\psi }(n)$ has a shape of $\left(1,\frac{M(M-1)}{2}\right)$, ${\hat{\psi }}_{ij}(n)=\psi_{ij}(n)$. The elements are then sorted in descending order according to their values, and only the largest *P* elements are retained, while all remaining elements are set to zero, and we have

(17) $$\hat{\psi }(n)=\left[\begin{array}{cccc} 0 & {\hat{\psi }}_{12}(n) & \cdots & 0\\ 0 & 0 & \cdots & {\hat{\psi }}_{2M}(n)\\ \vdots & \vdots & \ddots & \vdots \\ 0 & 0 & \cdots & 0 \end{array}\right]$$

where among the $M^{2}$ elements of matrix $\hat{\psi }(n)$, only *P* elements are nonzero, representing the pruned weights. Accordingly, the pruned GCC-PHAT can be expressed as

(18) $$G(w)=\hat{\psi }(n)W(w)\Phi (w)$$

where most elements in matrix $\hat{\psi }(n)$ are zero, and only *P* elements in Φ(w) and W(w) need to be computed. As a result, a substantial amount of computation is eliminated, reducing the computational cost to *n*/*M*^2^ of the original complexity. Meanwhile, during the DOA estimation process, the influence of unreliable microphone pairs is effectively suppressed, thereby reducing error propagation.

**Figure 3:**
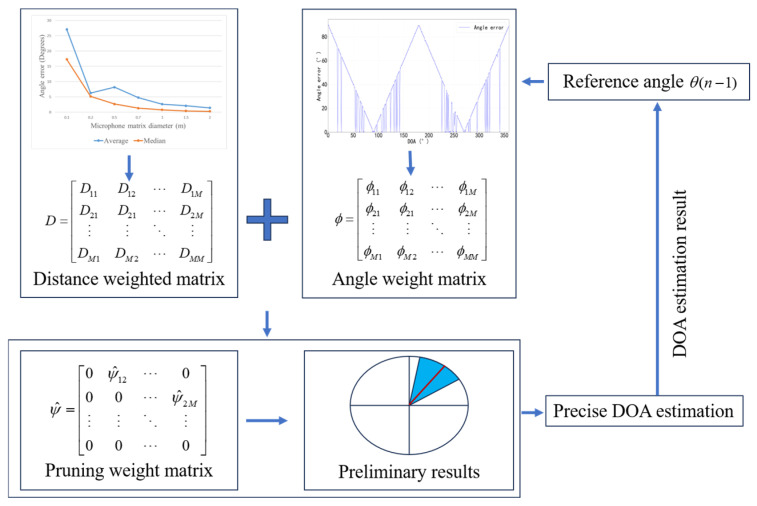
Schematic diagram of the dynamic pruning-based GCC-PHAT DOA estimation algorithm.

As illustrated in Figure 3 and Algorithm 2, the workflow of the weighted pruning GCC-PHAT-based DOA estimation algorithm can be summarized as follows:

**Algorithm** **2**: Target DOA estimation based on dynamic pruning GCC-PHAT**Input**: DOA estimation angle θ(n−1) at time (*n* − 1)-th frames, pruning retention number *P*.1: Generate distance weight matrix *D* based on the array.2: **If** *n* ≠ 1:  Using the DOA estimation result of (*n* − 1)-th frame as the estimated angle *θ*(*n* − 1) of the  incident sound source, generate an angle weight matrix *ϕ*(*n*), integrating *ϕ*(*n*) and *D*  to generate pruning weight matrix $\hat{\psi }(n)$.3: **Else**:  In the initial DOA estimation, randomly select *P* microphone pairs to generate $\hat{\psi }(1)$.4: Perform multi-microphone GCC-PHAT DOA estimation based on pruning recursion using$\hat{\psi }(n)$, and obtain the result $\hat{\theta }(n)$.5: Receive the DOA estimation result $\theta_{n}^{music}$ of the recursive MUSIC algorithm as the incident angle*θ*(*n*) of the sound source in the (*n* + 1)-th frame.**Output**: Search range for MUSIC algorithm in *n*-th frame $\left[\hat{\theta }(n)-\zeta ,\hat{\theta }(n)+\zeta \right]$.

### 3.4. Adaptive Forgetting Factor-Based Recursive MUSIC DOA Estimation Algorithm

In this section, a recursive MUSIC algorithm with an adaptive forgetting factor is proposed, where the adaptation is driven by the angular rate of change of the target DOA. In this approach, the subspace update at each iteration is governed by an instantaneous reconstruction error vector $e\left(n\right)$, which reflects variations induced by changes in the estimated DOA. The magnitude of $e\left(n\right)$ is used to adaptively adjust the forgetting factor $\lambda \left(n\right)$, thereby controlling the update of the subspace basis vectors $w\left(n\right)$. This mechanism establishes a feedback pathway of the following form: θ(n) variation → residual term $e\left(n\right)$ change → the subspace basis $w\left(n\right)$ adaptive update, resulting in a self-adjusting recursive MUSIC algorithm.

#### 3.4.1. Analysis of Frame Accumulation

The core of the conventional MUSIC algorithm lies in constructing the signal covariance matrix and decomposing the signal space into signal and noise subspaces. In practical applications, the covariance matrix *R* cannot be obtained directly and must instead be estimated from a finite number of snapshots via block accumulation:

(19) $$\hat{R}=\frac{1}{N}\sum_{i=1}^{N}x(i)x^{H}(i)$$

where $\hat{R}$ denotes the sample covariance matrix obtained via block-wise temporal accumulation, and *N* represents the time domain data samples.

The adaptive forgetting factor plays a crucial role in updating the exponentially weighted sample covariance matrix *R* in the MUSIC algorithm. Specifically, at time n, *R* can be expressed as follows [23]:

(20) $$R\left(n\right)=\lambda R(n-1)+x\left(n\right)x^{H}\left(n\right)$$

where $x\left(n\right)\in {\mathbb{C}}^{M\times 1}$ denotes the array observation vector, $R(n)\in {\mathbb{C}}^{M\times M}$ represents the sample covariance matrix of the array observations, and λ∈(0,1] is the forgetting factor. When *λ* is fixed and under the assumption that the observation vector is stationary, the equivalent time window *L*_eff_ can be expressed as

(21) $$L_{\mathrm{eff}}\approx \frac{1}{1-\lambda }$$

Since directly performing eigenvalue decomposition on *R* incurs high computational complexity, it is not explicitly computed within the OPAST recursive framework. Instead, the principal subspace is recursively approximated without explicitly constructing *R* by introducing a subspace basis matrix $W\left(n\right)$, a low-dimensional inverse correlation matrix $C\left(n\right)$, and a gain vector $G\left(n\right)$. Specifically [23],

(22) $$G(n)=\frac{C(n-1)y(n)}{\lambda +y^{H}(n)C(n-1)y(n)}$$

where $G\left(n\right)$ denotes the recursive gain vector, and $y\left(n\right)$ represents the projection of the current sample onto the subspace, i.e., the coordinates of the data in the signal subspace. $C\left(n-1\right)$ is the inverse correlation matrix in the low-dimensional projected space [23].

(23) $$C(n)=\frac{1}{\lambda }[C(n-1)-G(n)y^{H}(n)C(n-1)]$$

From Equations (22) and (23), it can be observed that the forgetting factor λ governs the system response speed by controlling the weighting of historical data. It also regulates the updates of $C\left(n\right)$ and $G\left(n\right)$, thereby indirectly influencing the evolution of *W*.

However, in practical scenarios, $x\left(n\right)$ is often nonstationary and varies over time. A larger forgetting factor λ can achieve higher steady-state accuracy but results in slower dynamic response. Conversely, a smaller λ improves tracking speed but increases sensitivity to noise and leads to larger steady-state errors. Therefore, a fixed forgetting factor cannot simultaneously balance steady-state accuracy, dynamic responsiveness, and noise robustness.

**Figure 4:**
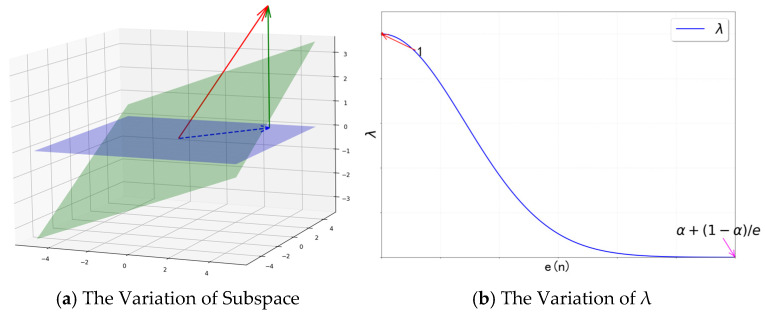
Schematic illustration of subspace and *λ* variation.

As shown in Figure 4a, in the joint OPAST–MUSIC DOA estimation framework based on subspace recursion, the blue plane represents the previous subspace, while the green plane denotes the updated subspace. The red arrow indicates the new observation vector, the blue dashed arrow corresponds to its projection onto the subspace, and the green arrow represents the orthogonal error vector.

If the new data $x\left(n\right)$ lies entirely within the previous subspace, then $e\left(n\right)\;=\;0$, and no subspace update is required. Conversely, if $x\left(n\right)$ does not lie in the previous subspace, $e\left(n\right)\neq 0$, and the subspace must rotate toward the direction of the error vector, resulting in the updated subspace represented by the green plane.

The adaptive forgetting factor λ driven by the residual vector $e\left(n\right)$ can effectively improve subspace tracking performance in dynamic target scenarios. The residual $e\left(n\right)$ characterizes the mismatch between the current observation and the existing signal subspace model, and its energy $∥e\left(n\right){∥}^{2}$ reflects variations in the statistical structure of the array signals.

When the target direction changes or the signal subspace drifts, $∥e\left(n\right){∥}^{2}$ increases significantly. In this case, the adaptively adjusted forgetting factor decreases accordingly, accelerating the forgetting of historical data and enhancing the sensitivity of subspace updates to new observations. Conversely, when the target state is stable, the residual energy remains small, leading to a larger forgetting factor, which allows the algorithm to retain longer-term historical information and improve the stability of covariance estimation.

Therefore, the adaptive λ based on $e\left(n\right)$ dynamically adjusts the effective temporal memory length according to the subspace matching degree, achieving a balance between fast tracking of dynamic targets and stable estimation. It improves both the accuracy and robustness of dynamic sound source DOA estimation.

Therefore, it is necessary to determine the attenuation of historical samples according to the current system state, thereby forming a dynamic window control mechanism. Based on this idea, an adaptive mechanism is proposed in which the forgetting factor λ is adjusted in real time according to the residual term $e\left(n\right)$.

#### 3.4.2. Adaptive Forgetting Factor Based on the Instantaneous Reconstruction Error Vector

For the adaptive forgetting factor λ, the instantaneous reconstruction error vector $e\left(n\right)$ provides an effective measure of the current subspace mismatch. When the subspace estimate is accurate, the magnitude of |e(n)| approaches the noise power level. However, when changes in the source incident angle cause variations in the observation vector $x\left(n\right)$, the value of |e(n)| increases significantly. Therefore, an adaptive mechanism for λ based on $e\left(n\right)$ enables automatic switching between dynamic tracking and steady-state estimation in response to variations in the source direction.

**Figure 5:**
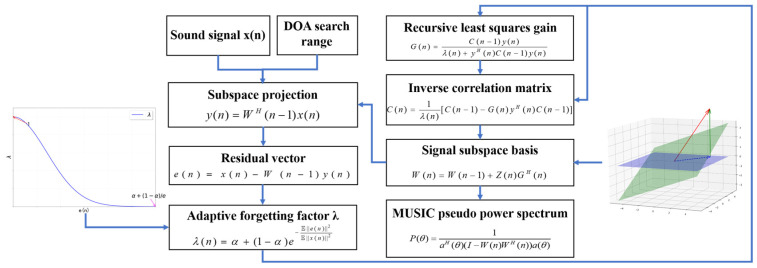
Schematic diagram of the adaptive forgetting factor-based OPAST-MUSIC algorithm.

As shown in Figure 5, the adaptive forgetting factor λ influences the update of the subspace *W* by affecting *C*(*n*) and *G*(*n*), thereby indirectly impacting the pseudo spectrum of the MUSIC algorithm. As indicated in Equation (23),

(24) $$\lambda (n)=\alpha +(1-\alpha )e^{-\frac{E||e(n)|{|}^{2}}{E||x(n)|{|}^{2}}},\;\alpha \in [0,1)$$

where α denotes a user-defined variable that can be designed according to specific requirements. The term *e*(*n*) represents the component of the covariance matrix that is not captured by the current subspace representation,

(25) $$e(n)=x(n)-\hat{x}(n)=(I-w(n-1)w^{H}(n-1))x(n)$$

where $\hat{x}(n)$ denotes the orthogonal projection of x(n) onto the current signal subspace.

#### 3.4.3. Analysis of Subspace Evolution

From Equation (25), it follows that 0≤||e(n)||≤||x(n)||. Let us define

(26) $$A(n)=\frac{E||e(n)|{|}^{2}}{E||x(n)|{|}^{2}},\;0\leq A(n)\leq 1.$$

According to the standard assumptions of the MUSIC algorithm, the noise across all channels is identical, mutually uncorrelated, and exhibits equal power in all directions, i.e., the noise power is $\delta^{2}I_{M}$. Subspace variation, in this context, refers to the rotation of the signal subspace. For any unitary matrix *Q*, it holds that

(27) $$Q^{H}(\delta^{2}I_{M})Q=\delta^{2}I_{M}$$

The identity matrix is invariant to rotation, implying that the noise component does not change with subspace variation. It follows that, in Equation (26), the value of *A*(*n*) is proportional to the angle of subspace change $\eta_{i}$; therefore, during the subspace recursion process, a faster subspace variation results in a larger value of *A*(*n*).

Specifically, in the subspace recursion process of OPAST, if the subspace remains unchanged or is accurately tracked, the value of $\eta_{i}$ approaches zero, and only the noise remains in the numerator of Equation (26), resulting in a minimal value of *A*(*n*).

When variations in the source incident angle lead to changes in the signal subspace, $\eta_{i}$ increases accordingly, causing an increase in *A*(*n*). Furthermore, if the source direction changes rapidly, resulting in significant subspace variation or tracking errors, $\eta_{i}$ approaches π2, leading to the maximum value of *A*(*n*).

It can therefore be observed that, as the source incident angle varies, the value of $\eta_{i}$ increases, leading to an increase in the numerator of *A*(*n*), while the denominator remains unchanged, and A(n)∈[0,1]. Accordingly, Equation (24) can be simplified as

(28) $$\lambda (n)=\alpha +(1-\alpha )e^{-A(n)},\;A(n)\in [0,1]$$

It is assumed that If we assume

(29) $$\chi (n)=e^{-A(n)},\;\chi \in [e^{-1},1]$$

where *A*(*n*) varies with *e*(*n*). From the previous analysis, the value of *A*(*n*) is positively correlated with *e*(*n*), i.e., it is positively related to the rate of change of the incident angle. Consequently, χ(n) is negatively correlated with the variation of *A*(*n*), and, thus, also negatively correlated with *e*(*n*). Moreover, in Equation (28), λ∈[α+(1−α)e,1]. In the experiment, the value of α is set to 0.9.

As can be observed from Figure 4b, when the incident angle of the sound source signal changes more rapidly, the subspace tracking error e in the recursive process increases. Consequently, the value of A approaches 1, χ decreases, and λ moves close to α+(1−α)/e. The equivalent frame accumulation window $L_{\mathrm{eff}}$ becomes shorter, enabling the recursive algorithm to respond faster and to place greater emphasis on the current acoustic signal. Conversely, when the incident angle varies slowly, the subspace remains nearly unchanged during recursion; e is close to 0, A is also close to 0, χ approaches 1, and λ approaches 1. In this case, the equivalent window $L_{\mathrm{eff}}$ lengthens, the algorithm responds more slowly, and more weight is assigned to the historical signal. Through this mechanism, λ is adaptively adjusted according to the variation of the incident angle and the resulting change in *e*.

## 4. Experimental Results

To comprehensively and objectively evaluate the performance of the proposed DOA estimation algorithm in complex vehicular acoustic environments, a multi-level experimental validation framework is designed, spanning from theoretical simulations to real-world deployment.

Section 4.1 conducts spectral analysis on the sound signal, followed by a simulation of the DOA algorithm to verify its absolute static accuracy, compare key parameters, and analyze its time complexity. To further evaluate its generalization capability, Section 4.2 conducts comparative experiments using the LOCATA dataset, verifying the algorithm’s fundamental processing performance on real acoustic signals under standardized benchmarks. Finally, Section 4.3 reports experiments conducted in real-world scenarios, where practical deployment performance is validated using measured data.

### 4.1. Sound Spectrum Analysis and Simulation Experiment

**Figure 6:**
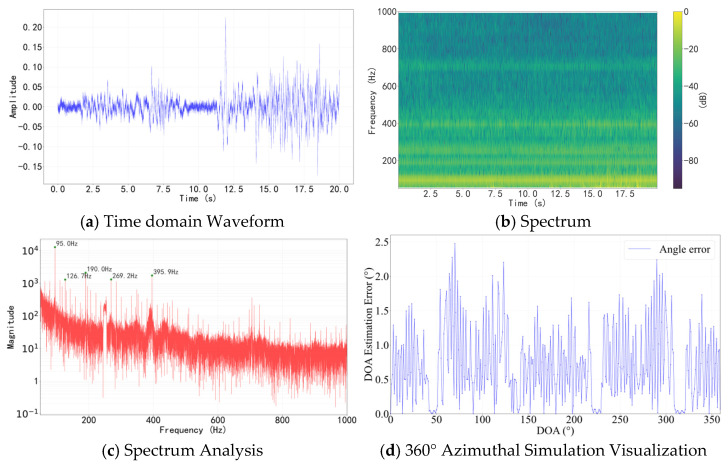
Signal waveform of the source signal and 360° DOA simulation.

The time domain waveform of the audio signal is shown in Figure 6a. As shown in Figure 6b, the sound source exhibits significant broadband nonstationary characteristics, with dominant energy mainly distributed below 500 Hz and maximum energy concentrated in the low-frequency band below 200 Hz. This frequency domain clustering phenomenon is highly consistent with the physical radiation mechanism of heavy-duty trucks, which is mainly composed of three parts: the power mechanism, the intake and exhaust system, and the walking transmission system. Based on these observations, the algorithm focuses on the identified core frequency bands to maximize the SNR of the effective components.

As shown in Figure 6c, the sound signal contains various components. The frequency of the peak signal energy is 95 Hz, with 190 Hz being its second harmonic. For narrowband algorithms, such as MUSIC and OPAST-MUSIC, the 190 Hz signal is used as the reference frequency, and the positive and negative 5 Hz signals near the reference frequency are preserved through preprocessing to transform the original broadband signal into a narrowband signal for DOA estimation. In the two-stage algorithm, the GCC-PHAT stage primarily exploits broadband signals dominated by low-frequency components, whereas the OPAST-MUSIC stage mainly relies on narrowband acoustic sources associated with higher-frequency harmonic components around 190 Hz.

In this section, simulation experiments are conducted using semi-real acoustic sources, with the simulation environment implemented in Python 3.13. The microphone array is configured as a planar cross-shaped array with a radius of 0.43 m, consisting of four uniformly distributed microphones. This large aperture design ensures a low-frequency response capability and provides high angular resolution.

In the simulation, the sound source is placed at a distance of 50 m from the microphone array. The recorded vehicle audio is combined with additive Gaussian white noise to control the SNR. To comprehensively and rigorously evaluate the spatial isotropy and angular resolution limits of the proposed algorithm over the entire azimuth domain, a panoramic numerical simulation framework is established. Independent experiments are conducted at 1° intervals around the array (a total of 360 trials), enabling the characterization of the theoretical error lower bound and noise-robust convergence behavior under extremely low-SNR conditions. For a thorough performance comparison, four representative algorithms are considered: GCC-PHAT, SRP-PHAT, MUSIC, and OPAST.

With respect to the key parameter—the number of pruned microphone pairs—comparative analyses are conducted both in the simulations presented in this section and in the dataset-based experiments in Section 4.2. These evaluations aim to investigate the impact of the pruning strategy on DOA estimation performance and computational efficiency. To objectively and multidimensionally quantify performance differences among algorithms, a comprehensive evaluation framework is introduced, comprising three core statistical metrics:

Mean Absolute Error (MAE): Measures the overall estimation bias across the global test set, reflecting the average lower bound of DOA estimation accuracy.

Median Absolute Error (MedAE): Evaluates the central tendency of the error distribution. This metric effectively suppresses the influence of extreme outliers, providing a more robust reference for baseline system performance.

Standard Deviation of Error (STD Error): Quantifies the dispersion and variance of estimation errors across different angles, directly reflecting the algorithm’s tracking smoothness and estimation consistency when handling signals from varying spatial directions.

#### 4.1.1. 360° Azimuthal Simulation

In the simulation environment, the receiving microphone array is statically deployed at the origin of the coordinate system. A virtual broadband sound source is positioned in the far field at a distance of 50 m from the array to approximate the plane wave incidence.

To eliminate systematic spatial bias associated with specific incident angles, a high-resolution spatial scanning strategy is employed, as illustrated in Figure 6d. The true azimuth angle of the source, denoted as $\theta_{true}$, is exhaustively scanned over the entire horizontal plane $\Theta \in \left[0{}^{\circ},359{}^{\circ}\right]$ without angular gaps, with a scanning resolution of Δθ=1°. This configuration results in 360 discrete azimuthal positions, at each of which an independent broadband acoustic observation is generated and processed for DOA estimation.

After completing exhaustive evaluations over 360 discrete angles, all estimated outputs across the spatial domain are globally aggregated to obtain a comprehensive error distribution model. This enables a macroscopic statistical assessment of the overall DOA estimation performance under full azimuth coverage.

The final results are summarized in Table 1, which presents a comparative evaluation of all algorithms under SNR conditions ranging from −15 dB to 5 dB. As shown in the table, at −15 dB, the DOA estimation error of all algorithms exceeds 20°, indicating that reliable operation is not achievable under such extremely low-SNR conditions.

At −10 dB, all algorithms except GCC-PHAT achieve an MAE below 5°. As the SNR increases, the estimation accuracy of all methods improves progressively. When the SNR reaches 0 dB, all algorithms except OPAST achieve an MAE below 1°, with the MedAE also less than or equal to 1°. At 5 dB, all algorithms achieve errors below 1°, demonstrating high-precision performance.

However, significant differences are observed in computational efficiency. The metric “Time” represents the average processing time per frame and reflects the computational complexity of each algorithm. Lower complexity corresponds to shorter processing time. Among the evaluated methods, GCC-PHAT exhibits the lowest computational cost, while both OPAST and the proposed algorithm operate within 10 ms per frame. In contrast, SRP-PHAT requires approximately 62 ms per frame on average, indicating substantially higher computational complexity.

In the static DOA estimation experiments, each angle is measured independently, eliminating the need to exploit temporal dependencies between adjacent signal frames. Consequently, the proposed algorithm, which leverages historical DOA estimates, cannot fully realize its potential advantages in this setting, resulting in a slight reduction in estimation accuracy compared with its performance in dynamic scenarios. Nevertheless, the proposed algorithm achieves estimation accuracy comparable to that of the other methods.

#### 4.1.2. Experiment on the Number of Retained Microphone Pairs in Pruning

For the key parameter—the number of retained microphone pairs after pruning *P*—Table 2 presents the results under different pruning levels and SNR conditions. Since the algorithm fails to operate reliably at −15 dB, the analysis starts from −10 dB.

At −10 dB, the DOA estimation accuracy is positively correlated with the number of microphone pairs: as the number of retained pairs increases, both the MAE and the STD Error decrease progressively. However, when the SNR is greater than or equal to −5 dB, the estimation accuracy is no longer positively correlated with the pruning level, and increasing the number of microphone pairs does not yield further performance improvements. In this regime, the effective contribution of microphone pairs becomes saturated.

Meanwhile, the computational cost continues to increase with the number of microphone pairs, leading to longer processing times. Therefore, under high SNR conditions, more aggressive pruning (i.e., fewer microphone pairs) can be applied to enhance real-time performance. In contrast, under low-SNR conditions, reducing the pruning level (i.e., retaining more microphone pairs) can improve DOA estimation accuracy.

#### 4.1.3. Comparison of Time Complexity

Assume that the array consists of M microphones, the number of frequency domain sampling points is F, the number of retained microphone pairs after pruning is *P*, the spatial search grid size is G, the restricted search grid size is g with g≤G, the number of snapshots is K, and the signal subspace dimension is d<M. Then, the computational complexity of various algorithms can be expressed in Table 3.

The computational cost of the GCC-PHAT algorithm is primarily concentrated in the estimation of cross-spectral density in the frequency domain and the inverse transformation. For each channel, a fast Fourier transform (FFT) must be performed, followed by cross-spectrum computation and PHAT weighting for all microphone pairs. Since the number of microphone pairs is $O\left(M^{2}\right)$, and each pair requires frequency and time domain transformations of complexity $O\left(F\log F\right)$, the overall computational complexity can be expressed as $O\left(MF\log F+M^{2}F\log F\right)$.

The SRP-PHAT algorithm extends GCC-PHAT by introducing a spatial grid search mechanism. Its computational complexity consists of two main components. The GCC-PHAT preprocessing stage has a complexity of $O\left(MF\log F+M^{2}F\log F\right)$, while the spatial scanning stage incurs an additional complexity of $O\left(GM^{2}\right)$. When the spatial resolution is high (i.e., large G), the computational burden is dominated by the spatial scanning term, resulting in a significant increase in computational cost for high-precision DOA estimation tasks.

The computational complexity of the MUSIC algorithm primarily arises from covariance matrix estimation, eigenvalue decomposition, and the subsequent spatial spectrum search. The corresponding complexities are $O\left(KM^{2}\right)$, $O\left(M^{3}\right)$, and $O\left(GM^{2}\right)$, respectively. Therefore, the overall computational complexity can be expressed as $O\left(KM^{2}+M^{3}+GM^{2}\right)$.

The OPAST–MUSIC algorithm reduces computational complexity by avoiding explicit eigenvalue decomposition of the covariance matrix. Its primary computational costs arise from subspace recursion and spatial spectrum search, with complexities of $O\left(Md\right)$ and $O\left(GMd\right)$, respectively.

The proposed method consists of two main components. In the GCC-PHAT stage, pruning reduces the number of microphone pairs to *P*. Assuming that no microphones are reused, the total number of microphones involved is *2P*, resulting in a computational complexity of $O\left(3PF\log F\right)$. In the OPAST-based stage, due to the restricted search region, the complexity of the spatial spectrum search is reduced to $O\left(gMd\right)$. g was defined as g=2ζ, and ζ was consistently set to 10° in the experimental evaluation. Therefore, the overall computational complexity of the proposed method is given by $O\left(3PF\log F+Md+gMd\right)$.

The computational complexity of the MUSIC algorithm is $O\left(M^{3}\right)$, while that of GCC-PHAT and SRP-PHAT is $O\left(M^{2}\right)$. Both OPAST and the proposed method exhibit a complexity of $O\left(M\right)$. In GCC-PHAT-based algorithms, only a small subset of microphone pairs is required for DOA estimation, i.e., $P<M<M^{2}$. Therefore, the pruned GCC-PHAT algorithm has significantly lower computational complexity than the full aperture GCC-PHAT approach. For the OPAST component, the search space is restricted, and the reduced grid size g is much smaller than G, resulting in substantially lower computational cost compared to the full search OPAST algorithm. Overall, it can be concluded that as the microphone array size increases, the proposed method exhibits increasingly pronounced advantages in both computational efficiency and real-time performance.

### 4.2. Experiments on the LOCATA Dataset

To further evaluate the generalization capability of the proposed algorithm and its performance on more complex array geometries, comparative experiments are conducted using the LOCATA dataset [42] in this section. Specifically, the *Benchmark2* setup is adopted, which employs a pseudo-spherical array consisting of 12 microphones integrated into the head of a NAO robot. Task 3 involves DOA estimation of a single moving speaker using a static microphone array. In this task, the speaker moves while speaking, requiring the algorithm to possess strong real-time tracking capability. Since the evaluated algorithms are nonlearning-based and do not require training, the eval subset of the dataset is directly used for testing.

In the LOCATA dataset, to facilitate a comprehensive comparison of DOA estimation performance, results are reported separately for the azimuth and elevation angles, while the processing time reflects the real-time capability of each algorithm. Ours-66 corresponds to the proposed framework that combines GCC-PHAT with OPAST-MUSIC, incorporating an adaptive forgetting factor λ. GCC+OPAST denotes the direct cascade of GCC-PHAT and OPAST-MUSIC. In pruning GCC+OPAST, the GCC-PHAT stage selects only six microphone pairs for time delay estimation, and the resulting estimates are subsequently processed by OPAST-MUSIC. As shown in Table 4, the proposed method (Ours-24) achieves the lowest error in azimuth estimation, whereas the minimum elevation error is obtained by SRP-PHAT. However, SRP-PHAT incurs significantly higher computational cost, resulting in poor real-time performance. Broadband methods, such as GCC-PHAT and SRP-PHAT, exhibit relatively lower DOA estimation errors, while narrowband approaches, including MUSIC and OPAST, show larger errors and fail to fully exploit the high-resolution capability of subspace-based methods.

The *Benchmark2* array consists of 12 microphones, forming a total of 66 microphone pairs. In this configuration, the impact of pruning on real-time performance becomes more pronounced. Therefore, the real-time performance under different pruning levels is systematically evaluated. Since at least three microphone pairs are required for 3D DOA estimation, the minimum pruning level is set to three pairs. When only three pairs are used, the estimation error is relatively large. Increasing the number to six pairs leads to a significant reduction in error. However, as the number of microphone pairs continues to increase, the improvement in accuracy becomes marginal. The best performance is achieved when 24 microphone pairs are retained. Beyond this point, further increasing the number of pairs results in a degradation of accuracy. This is because the contribution of additional microphone pairs becomes saturated, and pairs with larger estimation errors are introduced, which negatively affect the overall DOA estimation. Meanwhile, the computational time of the proposed method increases approximately linearly with the number of microphone pairs, rising from 34.71 ms to 99.21 ms. Cascading GCC-PHAT with OPAST-MUSIC can improve DOA estimation accuracy compared with using GCC-PHAT alone; however, the improvement comes at the cost of reduced real-time performance, as it increases the per-frame signal processing time. The OPAST-MUSIC method with an adaptive forgetting factor can effectively reduce DOA estimation errors, but it does not reduce the computational time required for signal processing.

The parameter *P* determines the number of microphone pairs retained after pruning. A smaller *P* reduces computational complexity and improves real-time performance, whereas a larger *P* preserves more spatial information and may improve robustness in noisy environments. For microphone arrays operating in relatively stable acoustic conditions, *P* can be initialized to the minimum value required for DOA estimation. When the SNR decreases, or the acoustic environment becomes more complex, a larger *P* may be adopted to improve estimation stability at the cost of increased computational load.

The search range ζ determines the angle search space of the second stage in our algorithm. A larger ζ improves robustness against coarse estimation errors but increases computational complexity, whereas a smaller ζ reduces processing time at the risk of missing the true DOA when the coarse estimate is inaccurate. Based on our experiments, ζ = 10° provides a good balance between estimation accuracy and computational efficiency in most scenarios. For situations with high real-time requirements, ζ may be reduced to 5°.

### 4.3. Real World DOA Estimation Experiments

**Figure 7:**
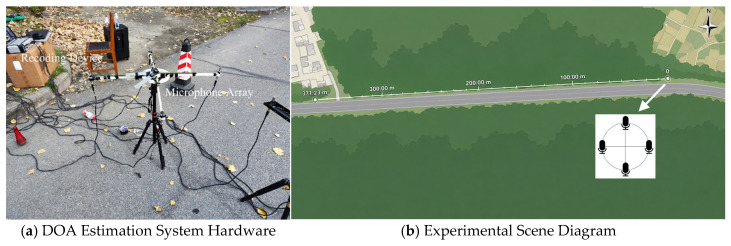
System hardware and experimental scene diagram.

To evaluate the practical performance of the proposed algorithm, the experimental platform is deployed in a real outdoor roadway environment. Compared with the idealized conditions in simulations and the controlled settings of benchmark datasets, this open outdoor scenario inherently introduces complex reflections and multipath effects, environmental wind noise, uncontrolled interfering sound sources, and background traffic noise. Such conditions provide a rigorous testbed for assessing the robustness of DOA estimation algorithms under real vehicular engineering constraints. In the experimental setup, the receiving microphone array is installed at the roadside to accurately emulate the operational perspective of a roadside sensing unit, as illustrated in Figure 7a.

The experiments are conducted on an outdoor roadway, where the sound source is a heavy-duty truck. The surrounding environment includes nearby forest and a parking area, with other vehicles passing along the road, as illustrated in Figure 7b. The microphone array is positioned at the roadside. Audio signals are synchronously acquired using four omnidirectional electret microphones with a 24-bit quantization resolution and a sampling frequency of 16 kHz. In the static experiments, distances are measured on the ground using a measuring tape, and the vehicle is positioned at a fixed distance from the array. After coming to a stop, the vehicle is kept in neutral gear while the engine is revved to generate acoustic emissions. During testing, both the vehicle and the array positions remain fixed. Different incident angles are emulated by rotating the microphone array. Considering the symmetry of the four-element cross-shaped array, only four representative angles—315°, 330°, 345°, and 360°/0°—are evaluated.

#### 4.3.1. Static Target DOA Estimation Experiments

For the calibration of spatial DOA estimation accuracy, a relative testing strategy based on source anchoring and array rotation is adopted. While keeping the physical position of the vehicle and its acoustic excitation state unchanged, the normal direction of the microphone array (i.e., the reference 0° direction) is manually rotated in equal angular steps relative to the true azimuth of the vehicle. Specifically, considering the symmetry of the array, four representative discrete relative angles—[315°, 330°, 345°, and 0°/360°]—are selected as ground truth labels. At each predefined array orientation, multiple segments of real engine noise signals, each lasting 20 s, are continuously recorded.

Table 5 presents a detailed comparison between the proposed algorithm and several baseline methods in a real-world static roadside experimental environment. The evaluation metrics include MAE, MedAE, and per-frame execution time, aiming to comprehensively assess the practical performance and engineering applicability of each algorithm.

In terms of DOA estimation accuracy and robustness, classical subspace-based methods, such as MUSIC and OPAST-MUSIC, theoretically offer high-resolution performance. However, under broadband sound sources and in the presence of large-aperture effects and real-world road multipath noise, these methods are prone to generating spurious peaks in distant angular regions. As a result, their MAE and MedAE degrade to some extent and in some cases become even larger than those of GCC-PHAT and SRP-PHAT-based methods. The proposed hybrid framework integrates the robustness of GCC-PHAT with the high resolution capability of subspace-based techniques, achieving consistently lower MAE and MedAE. The STD reflects the overall stability of angle estimation. As shown in the table, MUSIC and SRP-PHAT exhibit more concentrated frame-wise angle distributions, indicating lower variance in angular tracking and higher stability in DOA estimation. In contrast, the OPAST-MUSIC algorithm shows a larger STD, indicating reduced stability. The proposed method achieves the lowest MAE across all four angles while also obtaining a smaller STD error, demonstrating that it not only provides higher estimation accuracy but also maintains superior stability in DOA tracking. Merely cascading GCC with OPAST provides only a marginal accuracy gain over GCC-PHAT and still exhibits significantly degraded performance compared to the refined method.

**Figure 8:**
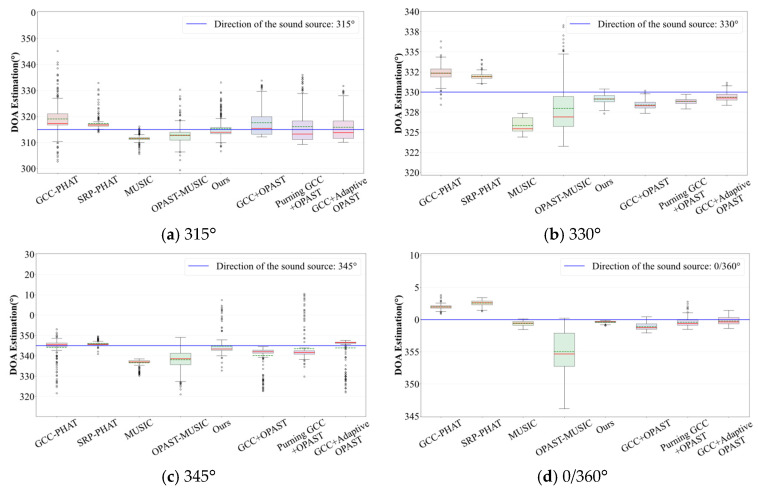
All frame angle distributions.

To more intuitively illustrate the DOA estimation results across all frames, Figure 8 presents box plots showing the distribution of estimates over time. In the figure, the green dashed line and red solid line represent the mean and median of the estimated angles across all frames, respectively. The circular markers outside the whiskers indicate outlier frames with relatively large estimation errors, while the blue solid line denotes the ground truth labels, i.e., the true incident angles of the sound source. As shown in the figure, the SRP-PHAT algorithm achieves excellent estimation accuracy and overall stability, with few outliers and results that closely align with the true angles. The MUSIC algorithm exhibits larger angular deviations but fewer outliers, indicating relatively stable overall behavior. In contrast, the proposed method achieves the best accuracy and stability among all compared approaches, with smaller estimation errors and more consistent performance across frames. In general, the two-stage algorithms demonstrate superior performance compared with the single-stage algorithms. Among all two-stage approaches, the Ours algorithm achieves the best balance between accuracy and robustness, yielding the lowest DOA estimation errors while maintaining the highest stability, as evidenced by its reduced performance fluctuations across different test scenarios.

Another key distinction lies in the computational efficiency of the algorithms. Table 6 reports the average execution time per frame for each method. Among all compared algorithms, GCC-PHAT requires the least computation, with an average of 2.33 ms per frame. The proposed method requires 2.91 ms per frame. MUSIC and OPAST-MUSIC exhibit comparable computational costs, both remaining below 10 ms per frame. In contrast, SRP-PHAT demonstrates the worst real-time performance, requiring 19.49 ms per frame, due to the need for spatial grid search after computing GCC-PHAT over all microphone pairs. Notably, the proposed method achieves better real-time performance than both MUSIC and OPAST-MUSIC. This is primarily because only three microphone pairs are utilized, and the OPAST-MUSIC search space is constrained to a localized region, significantly reducing computational overhead. The processing time difference of the two-stage algorithm is not significant. The observation suggests that the computational benefits of the pruning strategy are strongly dependent on the number of microphones in the array, with greater reductions in processing time expected for larger microphone arrays.

In summary, the proposed “dynamic pruning GCC-PHAT + adaptive OPAST-MUSIC” framework not only achieves superior DOA estimation accuracy, but also enhances system real-time performance and robustness in complex environments. In computation-constrained outdoor Internet of Things (IoT) and vehicular edge sensing scenarios, the proposed method demonstrates the best overall cost–performance ratio among the compared approaches.

#### 4.3.2. Dynamic Target DOA Estimation Experiments

In Section 4.3.1, sound sources with accurately measured distance and angular labels are deployed to rigorously evaluate different algorithms in terms of MAE, MedAE, STD, and average per-frame processing time. Additionally, box plots are used to present the macroscopic distribution of DOA estimates across all frames. These results constitute the quantitative foundation for assessing the DOA estimation accuracy of the proposed system in real-world scenarios.

**Figure 9:**
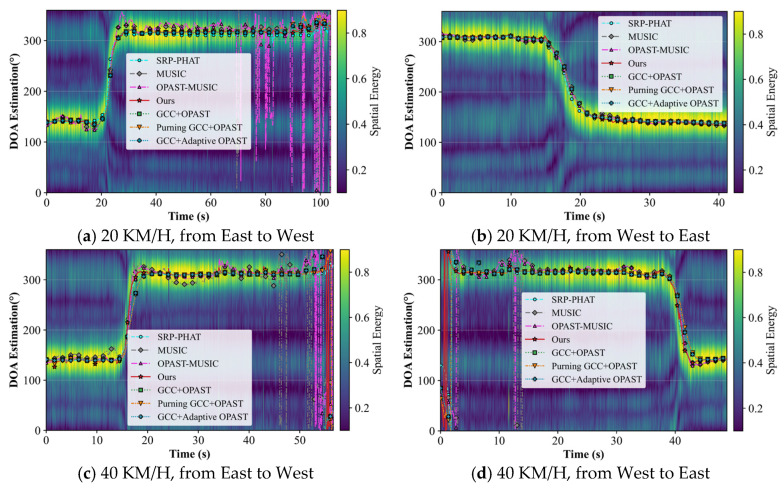

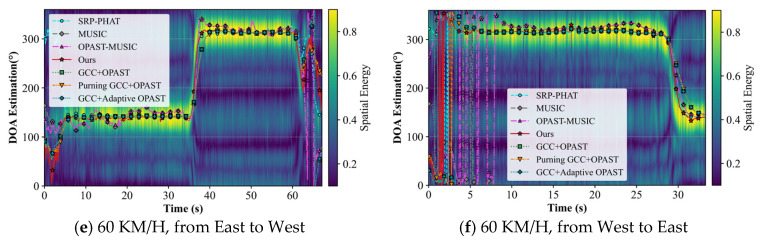
Dynamic target angle tracking results.

Due to the difficulty of precisely calibrating vehicle coordinates during high-speed motion, this section does not perform fine-grained alignment based on absolute localization errors. Instead, evaluation focuses on trajectory smoothness and consistency with acoustic energy distributions. To rigorously assess algorithm performance in real outdoor scenarios where explicit ground-truth coordinates are unavailable, Figure 9 presents an overlay of DOA trajectories estimated by different algorithms on the spatiotemporal energy pseudo-spectrum of GCC-PHAT. For this visualization, GCC-PHAT is excluded from the comparative set to serve as the reference energy map.

In this framework, the raw energy distribution without hard-decision filtering (background heatmap) objectively reflects the true physical acoustic field. The continuous high-energy ridges can be regarded as a reliable physical proxy for the true motion trajectory of the target vehicle, while the surrounding low-intensity regions correspond to environmental disturbances, such as wind noise and multipath reflections. As the vehicle moves, its relative position with respect to the array transitions from far to near, during which the azimuth angle initially changes slowly, then varies rapidly as it approaches the array, and finally changes gradually again as the vehicle moves away.

As indicated by the background map, the test environment is highly dynamic and heavily contaminated by noise. In addition to the target vehicle’s acoustic signal, complex environmental noise and other interfering sound sources are also present in the scene. At the beginning and end of the trajectory, the vehicle is far from the array, resulting in weak target signals while the background noise remains relatively constant; consequently, larger estimation errors are observed. During the tracking process, as shown in Figure 9a,d, the DOA estimates of the OPAST and MUSIC algorithms exhibit significant abrupt fluctuations. This indicates that, as narrowband methods, they are more susceptible to noise contamination. In contrast, the SRP-PHAT and the proposed method demonstrate more stable performance. Furthermore, as shown in Figure 9c,d, the trajectory of the proposed method is smoother than that of SRP-PHAT, indicating improved stability in DOA estimation.

In Table 7, MAE and MedAE denote the errors between the DOA estimates produced by each algorithm at each time instant and the angle corresponding to the maximum spatiotemporal energy in the background map shown in Figure 9. In Table 7, our algorithm achieves the smallest errors in most experiments, yielding lower values on both the MAE and MedAE metrics. This indicates that the DOA estimation results of our algorithm are closer to the angle with the maximum spatio-temporal energy, leading to a smaller overall error.

As can be observed in Table 7, the two-stage algorithms generally achieve higher accuracy than the single-stage algorithms, indicating that they effectively combine the advantages of both constituent methods. Among all evaluated approaches, the Ours algorithm attains the lowest errors in most experiments, achieving smaller MAE and MedAE values. These results demonstrate that the DOA estimates generated by the proposed algorithm are closer to the angle associated with the maximum spatial energy, thereby yielding lower overall estimation errors.

Throughout the entire testing period, the proposed algorithm consistently exhibits close agreement with the dominant energy ridge, yielding a smaller overall DOA estimation error than the other algorithms. This result provides a clear and compelling validation of the effectiveness of the proposed mechanism. Specifically, the dynamic GCC-PHAT pruning strategy acts as a form of soft spatial filtering, effectively removing severely corrupted microphone pairs prior to geometric fusion, thereby fundamentally eliminating the conditions that give rise to spatial pseudo-peaks. Meanwhile, the adaptive forgetting factor endows the MUSIC subspace with strong environmental awareness, enabling it to incorporate historical DOA information to maintain trajectory smoothness while simultaneously ensuring agile responsiveness to transient maneuvers. Overall, the proposed algorithm demonstrates superior engineering robustness and reliable real-time tracking performance in unstructured and complex acoustic environments.

## 5. Conclusions and Discussion

This study investigates the problem of vehicle sound source DOA estimation in outdoor roadway environments, with a particular focus on the comprehensive performance of DOA algorithms in terms of accuracy, real-time capability, and robustness under complex conditions. Given that traditional methods often struggle to simultaneously satisfy multiple performance criteria in practical applications, this work proposes a DOA estimation approach that integrates dynamic pruning-based generalized cross-correlation with an OPAST–MUSIC framework incorporating an adaptive forgetting factor, aiming to achieve an effective trade-off among multiple performance metrics.

The proposed method dynamically prunes based on the current DOA and the shape of the microphone array, and adaptively adjusts the forgetting factor through changes in the residual term. While maintaining stable DOA estimation accuracy, the method effectively reduces computational complexity and enhances robustness under low-SNR and dynamic-noise conditions. Compared with conventional GCC-PHAT, SRP-PHAT, MUSIC, and OPAST–MUSIC methods, the proposed approach demonstrates superior overall performance balance across multiple evaluation metrics.

Experimental results on simulated data, the LOCATA dataset, and real-world outdoor roadway measurements demonstrate that the proposed method operates stably under complex noise conditions and vehicular motion scenarios. On real data, it achieves lower DOA estimation errors compared with baseline methods, while simultaneously maintaining reduced computational complexity, higher real-time performance, and improved result stability. These findings indicate that the proposed approach exhibits strong feasibility and practical value for real-world engineering applications.

It should be noted that the proposed method still has certain limitations. The current study primarily focuses on single-source far-field DOA estimation scenarios, while its performance in multi-source DOA estimation and localization within complex spatial environments remains to be further investigated.

This work represents an initial step toward the development of a multi-node intelligent sensing network. Future research will focus on the following directions: (1) investigating collaborative processing among multiple array nodes within intelligent sensing networks to enable multi-target DOA estimation and localization; and (2) developing adaptive parameter tuning mechanisms to enhance the generalization capability of the algorithm across diverse environments.

## Figures and Tables

**Table 1:** 360° DOA simulation with a synthetic sound source.

Algorithm	−15 dB		−10 dB		−5 dB		0 dB		5 dB		Time (ms)
	MAE	MedAE	MAE	MedAE	MAE	MedAE	MAE	MedAE	MAE	MedAE	
GCC-PHAT	59.62	48.2	14.58	4.58	1.09	0.95	**0.7**	**0.57**	**0.55**	0.5	**2**
SRP-PHAT	32.73	**12**	**2.38**	**2**	**1.12**	**1**	0.77	0.6	0.62	1	62
MUSIC	**24.4**	19.5	4.78	4	1.75	1.5	0.82	0.75	0.72	**0.5**	27
OPAST-MUSIC	34.27	16.5	2.85	2.5	2.12	2	1.37	1.5	0.92	1	9
Ours	40.56	16	4.18	2	1.49	1.15	0.89	1	0.9	0.75	10

**Table 2:** Comparison of GCC-PHAT pruning numbers (*P*) under different SNR conditions.

SNR (dB)	*P*	2	3	4	5	6
	Time (ms)	9	9	10	10	11
−10	MAE	17.12	14.37	10	9.14	**7.14**
	MedAE	4	3	3	3	3
	STD Error	22.89	19.3	13.72	10.93	**8.41**
−5	MAE	2.08	**2.06**	2.12	2.14	2.13
	MedAE	2	2	2	2	2
	STD Error	1.29	**1.28**	1.34	1.32	1.31
0	MAE	1.89	1.89	1.89	1.89	1.89
	MedAE	1.7	1.7	1.7	1.7	1.7
	STD Error	1.91	1.21	1.21	1.21	1.21
5	MAE	0.88	0.88	0.88	0.88	0.88
	MedAE	0.75	0.75	0.75	0.75	0.75
	STD Error	0.65	0.65	**0.64**	0.65	0.65

**Table 3:** Comparison of algorithm time complexity.

Algorithm	Time Complexity
GCC-PHAT	*O*(*MF*log*F* + *M*^2^*F*log*F*)
SRP-PHAT	*O*(*MF*log*F* + *M*^2^*FlogF* + *GM*^2^)
MUSIC	*O*(*KM*^2^ + *M*^3^ + *GM*^2^)
OPAST-MUSIC	*O*(*Md* + *GMd*)
Ours	*O*(3*PF*log*F* + *Md* + *gMd*)

**Table 4:** Comparison of DOA estimation results on the LOCATA dataset.

Algorithm	Azimuth (°)		Elevation (°)		Time (ms)
	Mae	MedAE	Mae	MedAE	
GCC-PHAT	7.37	5.69	8.48	7.05	75.65
SRP-PHAT	9.93	7.46	**1.34**	**1.22**	283.46
MUSIC	11.12	8.6	4.06	3.58	44.07
OPAST-MUSIC	10.67	7.98	7.84	8.61	**26.77**
GCC+OPAST	7.16	6.03	6.94	6.2	100.55
Purning GCC+OPAST	6.76	6.04	5.41	4.61	44.41
Ours-3	13.26	10.57	4.5	3.32	34.71
Ours-6	6.88	5.80	5.68	4.64	39.01
Ours-12	6.41	5.81	5.19	4.55	45.45
Ours-24	**6.16**	**5.11**	5.1	4.12	55.90
Ours-48	6.28	5.25	5.12	4.1	79.27
Ours-66	6.24	5.23	5.07	4.03	99.21

**Table 5:** Comparison of DOA estimation results for static targets.

Algorithm	315°			330°			345°			0/360°		
	Mae	MedAE	STD	Mae	MedAE	STD	Mae	MedAE	STD	Mae	MedAE	STD
GCC-PHAT	4.06	2.29	6.59	2.35	2.25	0.95	0.83	**0.43**	4.98	1.98	1.97	0.38
SRP-PHAT	2.33	1.7	2.66	1.96	1.92	0.44	0.94	0.69	**1.02**	2.57	2.61	0.43
MUSIC	3.49	3.49	**1.43**	4.15	4.56	0.85	11.04	7.34	1.65	0.59	0.55	0.38
OPAST-MUSIC	2.14	2.25	3.84	2.03	3.09	3.14	6.92	6.41	5.08	0.98	11.27	3.06
Ours	**0.36**	1.01	4.2	0.87	0.86	0.54	**0.22**	1.63	4.99	0.38	**0.34**	**0.18**
GCC+OPAST	2.59	**0.43**	5.48	1.61	1.49	0.54	0.84	1.56	6.33	1.1	1.25	0.51
Purning GCC+OPAST	1.07	1.75	6.56	1.16	1.15	**0.38**	0.72	0.87	3.01	0.4	0.6	0.68
GCC+Adaptive OPAST	0.87	1.18	5.44	**0.63**	**0.74**	0.53	1.08	1.54	6.36	**0.22**	0.36	0.6

**Table 6:** Comparison of algorithm execution times.

Algorithm	GCC-PHAT	SRP-PHAT	MUSIC	OPAST-MUSIC	Ours	GCC+OPAST	Purning GCC +OPAST	GCC+Adaptive OPAST
Time (ms)	**2.33**	19.49	6.9	7.8	2.91	3.09	2.81	3.13

**Table 7:** Dynamic sound source tracking angle error in Figure 9.

Direction	From East to West						From West to East					
Speed (km/h)	20		40		60		20		40		60	
Angle Error (°)	MAE	MedAE	MAE	MedAE	MAE	MedAE	MAE	MedAE	MAE	MedAE	MAE	MedAE
SRP-PHAT	5.0	3.9	5.12	3.05	16.38	3.0	4.48	3.0	5.52	1.83	5.89	1.7
MUSIC	11.12	9.6	18.29	11.78	20.27	12.08	4.92	4.55	12.56	5.71	21.15	11.91
OPAST-MUSIC	19.11	11.12	9.26	5.89	19.2	11.61	4.17	3.83	11.08	6.11	18.36	11.93
Ours	**2.40**	2.29	2.17	**1.34**	**8.16**	**2.27**	**1.54**	**1.53**	**1.97**	**1.79**	**4.34**	2.17
GCC+OPAST	3.34	2.6	5.04	3.15	12.65	3.43	2.79	2.64	6.42	2.48	6.06	1.57
Purning GCC+OPAST	2.91	1.83	4.87	1.49	11.27	2.49	2.52	2.51	5.6	2.12	7.29	2.35
GCC+Adaptive OPAST	2.74	**1.77**	4.38	2.32	12.23	2.74	2.51	2.47	6.37	2.5	6.08	**1.56**

## Data Availability

Data is contained within the article.
